# Altered spawning seasons of Atlantic salmon broodstock transcriptionally and epigenetically influence cell cycle and lipid-mediated regulations in their offspring

**DOI:** 10.1371/journal.pone.0317770

**Published:** 2025-02-24

**Authors:** Takaya Saito, Marit Espe, Maren Mommens, Christoph Bock, Jorge M.O. Fernandes, Kaja Helvik Skjærven

**Affiliations:** 1 Institute of Marine Research, Bergen, Norway; 2 AquaGen, Trondheim, Norway; 3 CeMM, Research Center for Molecular Medicine of the Austrian Academy of Sciences, Vienna, Austria; 4 Nord University, Bodø, Norway; National Cheng Kung University, TAIWAN

## Abstract

Manipulating spawning seasons of Atlantic salmon (*Salmo salar*) is a common practice to facilitate year-round harvesting in salmon aquaculture. This process involves adjusting water temperature and light regime to control female broodstock maturation. However, recent studies have indicated that altered spawning seasons can significantly affect the nutritional status and growth performance of the offspring. Therefore, gaining a deeper understanding of the biological regulations influenced by these alterations is crucial to enhance the growth performance of fish over multiple generations. In this study, we investigated omics data from four different spawning seasons achieved through recirculating aquaculture systems (RAS) and sea-pen-based approaches. In addition to the normal spawning season in November (sea-pen), three altered seasons were designated: off-season (five-month advance, RAS), early season (two-month advance, sea-pen), and late season (two-month delay, sea-pen). We conducted comprehensive gene expression and DNA methylation analysis on liver samples collected from the start-feeding larvae of the next generation. Our results revealed distinct gene expression and DNA methylation patterns associated with the altered spawning seasons. Specifically, offspring from RAS-based off-season exhibited altered lipid-mediated regulation, while those from sea-pen-based early and late seasons showed changes in cellular processes, particularly in cell cycle regulation when compared to the normal season. The consequences of our findings are significant for growth and health, potentially providing information for developing valuable tools for assessing growth potential and optimizing production strategies in aquaculture.

## Introduction

The Atlantic salmon (*Salmo salar*) follows an anadromous life cycle, beginning in freshwater, migrating to seawater for most of its life, and returning to freshwater to spawn. In salmon aquaculture, the timing of spawning is adjusted to ensure year-round harvesting availability. This manipulation involves controlling abiotic factors, such as water temperature and photoperiod, to influence the spawning timing [1,2]. Research has shown that a sudden shift from long to short day lengths in summer promotes ovulation, while continuous light delays it [3]. Additionally, elevated temperatures can delay ovulation [4]. This knowledge has contributed to the development of a flexible salmon production system. As an example of this manipulation, along with detailed method descriptions, a recent study on Atlantic salmon demonstrated the ability to adjust spawning seasons by controlling temperature and light regimes after transferring the fish to freshwater [5]. Additionally, apart from the traditional open sea-based cages, a land-based approach utilising recirculating aquaculture systems (RAS) is employed to more accurately replicate the salmon’s natural lifecycle, including both saltwater gonad maturation and final freshwater maturation periods [6]. Despite the ongoing and successful practices of spawning season alterations in salmon aquaculture, recent studies have reported that these practices can impact the nutritional status of broodstock and eggs, leading to negative effects on the growth performance in the next generation [7,8].

While various nutrients influence the health condition and growth performance of Atlantic salmon [9–11], altered spawning seasons have appeared to affect the status of multiple metabolites and nutrients including micronutrients, amino acids, and lipid classes [7,8]. These nutrients are also closely associated with vital biological functions necessary for growth, such as one carbon (1C) metabolism, the citric acid cycle, and the Cahill cycle. Multiple micronutrients, including vitamin B12, folate, vitamin B6, and methionine, are integrated with the 1C metabolism [12,13], which regulates cellular methylation potential through S-adenosylmethionine (SAM) and S-adenosylhomocysteine (SAH), critical for physiological processes [14]. The citric acid cycle enables the catabolism of carbohydrates, fats, and amino acids, releasing stored energy crucial for growth and development [15], while the Cahill cycle facilitates the transport of amino groups and carbons between muscle and liver [16], both playing essential roles in various developmental processes. Amino acids are essential for growth and tissue development, in addition to being the building blocks of proteins [17], while lipids serve as major energy reserves and contribute to the formation of cell membranes, also supporting various developmental processes [18].

Beyond the importance of nutrients, understanding genetic influences provides invaluable insights from a molecular biology perspective. Recent studies have revealed that alterations in micronutrient levels within the feed can have transcriptomic effects on various biological pathways in salmon, including lipid metabolism, protein synthesis, and post-transcriptional regulation [19,20]. Moreover, an additional layer of information comes from the analysis of DNA methylation, which supports to reveal important epigenetic regulations. DNA methylation, as an epigenetic regulatory process, influences various cellular mechanisms, including cell stability, differentiation, and development, potentially in response to environmental stimuli [21,22]. Since DNA methylation is inherited during cell replication, it can result in long-term changes in epigenetic regulation [21,23].

The present study investigates the transcriptomic and epigenetic effects on offspring resulting from four different spawning seasons. These seasons include the natural sea-pen-based spawning in November, an accelerated RAS-based spawning occurring five months earlier, and two variations of sea-pen-based spawning: one with a two-month advance and another with a two-month delay relative to the natural spawning season. The main analysis of the study focuses on omics analysis of liver samples collected from start-feeding larvae at the end of the endogenous feeding phase. We selected the liver as our target tissue for several reasons. The liver serves as a key target organ for various metabolic reactions crucial to growth, and using start-feeding larvae helps eliminate the influences of exogenous feeding. Additionally, a zebrafish study showed that nutritional programming in parents may lead to epigenetic regulation associated with liver lipid accumulation and a fatty liver-like phenotype in the offspring [24]. Omics data in the liver are also available from other Atlantic salmon studies [19,20].

To uncover both transcriptomic and epigenetic impacts comprehensively, we employed RNA-sequencing (RNA-seq) for gene expression and reduced-representation bisulfite sequencing (RRBS) for DNA methylation analysis. Our findings suggest that gene expression and DNA methylation data can serve as powerful tools for assessing current and future growth performance. These tools could potentially benefit a wide range of nutritional programming studies and enhance our understanding of how aquatic species adapt to climate change.

## Methods and Materials

### Ethical considerations

The experiment adhered to the ARRIVE guidelines for design and reporting. Broodstock and offspring used in the study were obtained from AquaGen’s commercial production facility of Atlantic salmon at their breeding station in Kyrksæterøra, Norway.

As the samples were collected at the breeding station, the conditions and protocols applied in this experiment were identical to those used in commercial production, including certain confidential information protected under commercial interests. Throughout the sampling procedures for broodstock and larvae, anaesthetics were administered by the supplier’s instructions and compliance with both Norwegian and European legislation on animal research.

Formal approval from the Norwegian Animal Research Authority (NARA) was not required for this experiment. This exemption is because the experimental conditions fall under practices recognized as commercial animal husbandry, thereby exempting them from the European Convention on the protection of animals used for scientific purposes (2010/63/EU), specifically under Article 5d. Additionally, the Norwegian ethics board also approved the experiment by the Norwegian regulation on animal experimentation, § 2, 5a, d, for activities classified as “non-experimental husbandry (agriculture or aquaculture)” and “procedures in normal/common breeding and husbandry”.

### Experimental design and sampling

As the details of experimental design and sampling methods have been described elsewhere [7,8], we only present summarized descriptions here. The study utilized broodstock and offspring obtained from AquaGen’s breeding station in Kyrksæterøra, Norway. The female broodstock, sourced from the 15th generation, covered four spawning seasons: off-season, early, normal, and late seasons.

The off-season broodstock were reared in a land-based recirculating aquaculture system (RAS) in brackish (12‰ salinity), while the broodstock from the early, normal, and late seasons were raised in three open sea-based net pens with ambient photoperiod and temperatures. All broodstock were fed to satiation with broodstock feed (EWOS Breed 3500) until transferred to freshwater. In freshwater, the normal season broodstock matured naturally under ambient photoperiod and temperatures, whereas artificial abiotic factors like temperature and light were applied to regulate maturation for the other spawning seasons. Specifically, while the normal season broodstock were maintained at 6 °C with an 8-hour daylight cycle upon transfer to freshwater tanks in August, the early season broodstock were maintained by following the protocol outlined by Naeve and colleagues [25]. Details about the temperature and photoperiod used to postpone maturation in the off-season and late season groups are proprietary information owned by AquaGen AS. The off-season and early season broodstock were subjected to five- and two-month advance maturation, respectively, while the late season broodstock were subjected to a two-month delay. The fish received no further feeding after the transfer to freshwater. The maturation process in freshwater lasted for 109, 163, 120, and 166 days for off-season, early, normal, and late season broodstock, respectively, before spawning to obtain the next generation (Supplementary Table S1 in S1 File).

From each spawning group, five females from separate tanks were sacrificed using Benzoak (200 mg/L, ACD Pharmaceuticals AS). After stripping, each female’s growth measures, including body weights (n = 5 per spawning season, randomly selected), were recorded. Liver samples were dissected and flash frozen in liquid N2 for nutrient analysis. All oocytes were fertilised with cryopreserved sperm from two pooled males. Newly fertilized eggs at two degree days (2 d°) were flash-frozen in liquid N2 for nutrient analysis (Supplementary Table S1 in in S1 File). Degree days are calculated as the cumulative thermal exposure, obtained by multiplying the number of days by the temperature difference above the minimum growth temperature of 15°C for Atlantic salmon.

At the end of the endogenous feeding phase (start-feeding larvae 979–994 d°; Supplementary Table S1 in S1 File), 36 larvae from each broodstock were randomly selected, euthanised with an overdose of buffered tricane methanesulfonate (MS222, Pharmaq, Norway), and body weights (n = 180 per spawning season) were recorded. Additionally, 40 liver samples (two from each broodstock fish) were dissected from larvae and flash frozen in liquid N2 and used for either RNA or DNA analysis.

### Growth measure and nutrient analysis

We collected the data on body weights for broodstock and larvae from previous studies [7,8]. In addition, we obtained the data on selected nutrients and metabolites from the same studies, specifically vitamin B6 & B12, folate, S-adenosylmethionine (SAM), S-adenosylhomocysteine (SAH), cholesterol, the sum of six different lipids, glutamate, L-serine, L-lysine, L-alanine, L-glutamine, urea, and B-alanine [7,8]. The previous studies employed two method, biochemical approaches and LC-MS/MS, to measure folate content. In the present study, we used the total folate values from the LC-MS/MS method in those studies. For calculating total lipid values, the six lipids analysed included phosphatidylcholine, phosphatidylinositol, phosphatidylethanolamine, triacylglycerol, cholesterol, and sphingomyelin. To analyse the data from all four spawning seasons, we performed ANOVA followed by Tukey’s HSD test.

### DNA and RNA extraction

RNA extraction from 20 larvae livers (n = 5 for each spawning season) was carried out using the BioRobot EZ1 and EZ1 RNA Universal Tissue kit (Qiagen), followed by DNase treatment using the Ambion DNA-free DNA removal kit (Invitrogen, USA) according to their respective protocols. The RNA quantity was assessed using the NanoDrop ND-1000 Spectrophotometer (Nanodrop Technologies) and the Agilent 2100 Bioanalyzer with the RNA 6000 Nano LabChip kit (Agilent Technologies). For DNA isolation from 20 larvae livers (n = 5 for each spawning season), the DNeasy Blood & Tissue Kit (Qiagen, Cat. No. #69506) was used as per the manufacturer’s protocol. Liver samples underwent RNase A treatment (provided by the Qiagen kit, 50ng/ µ L, 10 min at room temperature) followed by proteinase K treatment (New England Biolabs, #8102S 20µg/ µ L, 1.5 h at 55°C). DNA was eluted in Milli Q water. DNA quantification was performed using the Qubit High Sensitivity Assay (Life Technologies #Q32854). Detailed methods for both DNA and RNA extraction were described elsewhere [19].

### Library preparation for RNA-seq and RRBS

Liver samples were sequenced at two different sequencing facilities for specific analyses. For RNA-seq, the liver samples were processed at the DeepSeq sequencing facility at Nord University, Bodø, Norway, and the library preparation was conducted using the NEBNext Ultra II Directional RNA Library Prep Kit for Illumina (New England Biolabs). Sequencing was performed on the NextSeq500 machine (Illumina). For RRBS, the liver samples were processed at the CeMM Biomedical Sequencing Facility, Vienna, Austria. The genomic DNA was extracted, digested by MspI, and bisulfite-converted before library preparation. The RRBS libraries were sequenced on Illumina HiSeq 3000/4000 instruments. Detailed methods of the library preparation for RNA-seq and RRBS were described elsewhere [19].

### Atlantic salmon genome and genomic annotation

The reference genome (ICSASG version 2) and RefSeq data (version 100) for gene annotation were downloaded from the NCBI web site (https://www.ncbi.nlm.nih.gov/assembly/GCF_000233375.1). In cases where gene symbols were either outdated or unavailable from RefSeq data (version 100), gene symbols from a newer version of RefSeq data (version 102) and UniProt [26] were used. The genome regions were annotated into intron, exon, three promoter regions, and flanking regions. Promoter regions were divided into three categories based on their distance from transcription start sites: P250 (1–250 bp), P1K (251–1000 bp), and P5K (1001–5000 bp). Flanking regions were defined as 5000 upstream from P5K and 10000 downstream from the 5’ end of the gene.

### Pre-processing of high throughput sequencing

For both RNA-seq and RRBS data, the same pre-processing procedures were followed as described in a previous study [19]. This involved trimming the reads using Cutadapt [27] and Trim Galore! (Barbraham Institute), aligning the trimmed reads to the reference genome using STAR [28] for RNA-seq and Bismark/Bowtie 1 [29,30] for RRBS with their default parameters. The mapped RNA-seq reads were quantified using featureCounts [31], while the mapped RRBS reads were processed by Bismark [29] for methylation calling and CpG site extraction. The samples were divided into four spawning season groups for further analysis: off-season, early, normal, and late seasons, and clustering analysis was performed using the factoextra package (https://CRAN.R-project.org/package=factoextra). RNA-seq counts were subjected to a variance stabilizing transformation (VST) using DESeq2 [32] before principal component analysis (PCA).

Raw reading data were uploaded to the repository of the Sequence Read Archive (SRA) on the NCBI web site and available under the accession numbers PRJNA680425 and PRJNA642998 for RNA-seq and RRBS, respectively.

### Differential gene expression analysis

Differentially expressed genes (DEGs) were identified using DESeq2 [32] with an adjusted p-value cut-off of less than 0.05. P-values were adjusted by the Benjamini-Hochberg procedure [33]. From the candidate DEGs, those with absolute log fold changes (LFCs) greater than or equal to 1.2 were selected as the final set of DEGs by using the lfcThreshold argument of the results function of DESeq2 [32]. Shrunken LFCs values, calculated by the normal shrinkage method provided by DESeq2 [32], were used for both heatmaps showing functional annotation results and scatter plots showing merged results between DNA methylation and gene expression differences.

### Functional annotation with KEGG

Over-representation analysis (ORA) was performed on the Kyoto Encyclopedia of Genes and Genomes (KEGG) database [34] using clusterProfiler [35]. Gene lists of DEGs identified from the three pair-wise comparisons against the normal season were used as input, and enriched pathways were determined based on adjusted p-values less than 0.05. P-values were adjusted by the Benjamini-Hochberg procedure [33].

### Methylation rate analysis

Mapped RRBS reads were filtered by methylKit [36], discarding reads with coverage less than or equal to 10 and above the 99.9th percentile. Additional RRBS data from two other studies were obtained for post-smolt and harvesting stages [19,20] and underwent the same pre-processing procedures to compare the distributions of methylation rates. Raw data of these RRBS sequencing data can be found in SRA on the NCBI site under the accession numbers of PRJNA680423 and PRJNA628740 for post-smolt and harvesting stages, respectively.

### Differential methylation analysis

Differentially methylated CpG sites (DMCs) were identified by methylKit [36], and Q-values were calculated using the sliding linear model (SLIM) method [37]. CpGs were identified as DMCs when Q-values were less than 0.01 and methylation differences were greater than or equal to 15%.

To filter out DMCs that were strongly affected by altered spawning seasons, we used the counts of DMCs per gene in two promoter regions: P250 and P1K. Genes were sorted by the count of DMCs in a descendant order, separately for P250 and P1K, and genes that contained at least three DMCs were selected for further analysis.

### Bioinformatics analysis

In-house R and Python scripts with Snakemake [38] were utilized for high-throughput sequence analysis, basic statistical analysis, and figure generation.

## Results

### Altered spawning seasons impacted the weight and nutritional status of offspring

**Fig 1:**
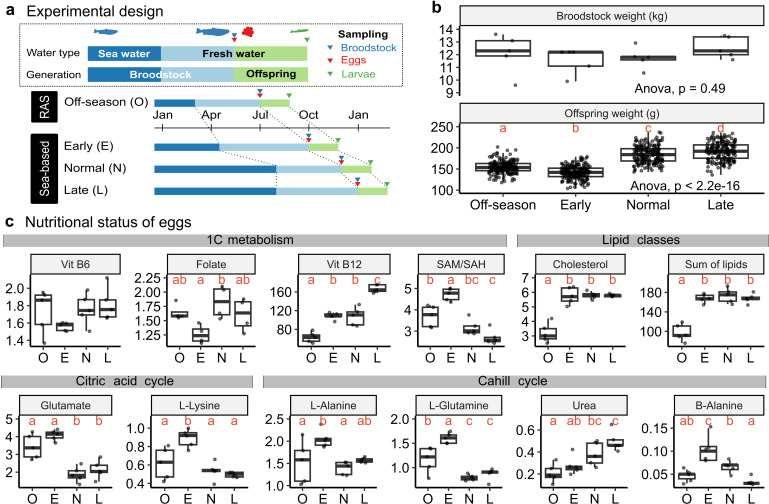
Experimental design, body weights, and nutritional status across spawning seasons. **a)** Schematic diagram illustrating four distinct spawning seasons: off-season (O), early (E), normal (N), and late (L). The off-season was conducted in RAS, while others were in a sea-based environment. Broodstock were transferred from seawater (blue bars) to freshwater (light blue bars) before spawning. Offspring were raised in freshwater until sampling (green bars). Triangles indicate sampling points: broodstock (blue), eggs (red), and larvae (green). **b)** Boxplots showing body weights of broodstock (n = 5) and larvae (n = 180) per season. Red letters represent Tukey’s HSD compact letter display after ANOVA tests (p < 0.05). **c)** Boxplots of 12 nutrients and metabolites in egg samples. Nutrients and metabolites are categorised into four groups: 1C metabolism, lipid classes, citric acid cycle, and Cahill cycle. Units: 1C metabolism group (Vitamin B6: mg/kg ww, Folate: mg/kg ww, Vitamin B12: µg/kg ww); remaining groups: lipid classes (mg/g ww), citric acid cycle (µmol/g ww), Cahill cycle (µmol/g ww). Red letters represent Tukey’s HSD compact letter display after ANOVA tests (p < 0.05).

The present study examines the effects on gene expression and DNA methylation patterns in the liver of offspring hatched from four different spawning seasons: an off-season based on a recirculating aquaculture system (RAS) and three sea-pen based seasons (early, normal, and late) (Fig 1a). Off-season spawning occurred in June, early season in September, and late season in January, relative to the normal spawning months of November (see detailed dates and degree days in Supplementary Table S1 in S1 File). The growth performance and nutritional status of the broodstock and offspring have already been assessed and discussed elsewhere [7,8]. Specifically, these preceding studies included analyses of liver and muscle nutrients in broodfish, newly fertilised eggs, and eye-stage embryos, as well as assessments of growth performance. In addition, growth performance was measured in start-feeding larvae. This section provides a summary of previous research, emphasizing the weight of broodfish and start feeding larvae, along with the status of selected nutrients in newly fertilised eggs. The primary focus of the present study, however, is on the omics analysis of start feeding larvae.

Broodstock weights showed no significant differences at spawning (upper part of Fig 1b), but offspring weights at the larvae stage (979 - 994 degree days) showed noticeable variation (lower part of Fig 1b). Specifically, off-season and early season larvae had lower weights compared to normal and late seasons (Supplementary Table S2 in S1 File). This trend was consistent with the observations in egg sizes, where eggs from off-season and early-season exhibited smaller sizes compared to the other two seasons (see the number of eggs per liter in Supplementary Table S1 in S1 File).

Altered spawning seasons widely impacted the nutritional status of offspring eggs, particularly regarding 1C metabolism, lipid classes, citric acid cycle, and Cahill cycle [7,8], but with varying patterns across nutrients and metabolites (summarised in Fig 1c and Supplementary Tables S3–S6 in S1 File). Previous studies also reported that altered spawning seasons affected the nutritional status of the broodstock in addition to their offspring [7,8]. Thus, altered spawning seasons significantly influenced the nutritional status of broodstock without impacting their weights, however, subsequently affecting offspring nutritional status and growth potential of their offspring.

### Early and late spawning seasons exhibited similar gene expression patterns when compared to the normal season

To investigate the impact of different spawning seasons, we performed gene expression analysis on 20 liver samples, which were divided into four groups (n = 5). We compared gene expression from the normal season to that from the other three altered spawning seasons (detailed statistics for each sample are provided in Supplementary Table S7 in S1 File).

**Fig 2:**
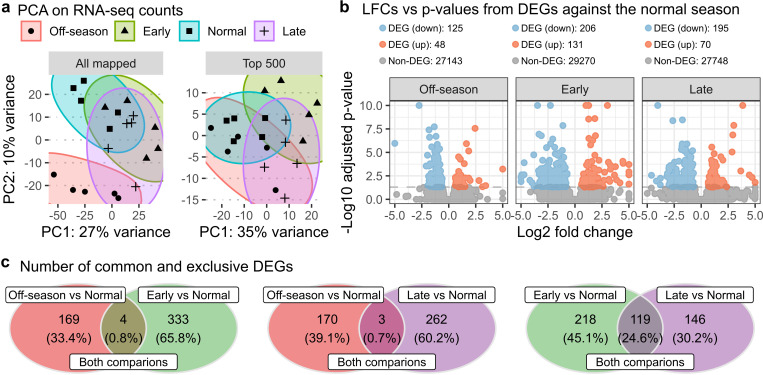
Gene expression analysis comparing altered spawning seasons to the normal season. **a)** PCA plots displaying clustering patterns of four spawning seasons—off-season (red), early (green), normal (blue), and late (purple)—using 20 RNA-seq samples. “All mapped” plot includes 45153 genes, while “Top 500” includes top 500 genes with high variance. **b)** Volcano plots showing differential expression analysis results for three pairwise comparisons: off-season vs. normal (Off-season), early vs. normal (Early), and late vs. normal (Late). Dots represent down-regulated DEGs (blue), up-regulated DEGs (red), and non-DEGs (grey). The counts of DEG and non-DEGs were provided in the legend. **c)** Venn diagrams illustrating overlaps of DEGs identified between two comparisons, indicating common DEGs as well as those exclusive to a single comparison.

Principal component analysis (PCA) differentiated the off-season from the early and normal seasons when considering all mapped genes (left part of Fig 2a). However, when focusing on the top 500 genes with high variances, the off-season moderately deviated from the early season but not notably from the normal season (right part of Fig 2a). In both cases, the late season overlapped partially with all other seasons.

In three pairwise comparisons for differential expression analysis against the normal season (off-season vs. normal, early vs. normal, and late vs. normal), we identified 173, 337, and 265 differentially expressed genes (DEGs), respectively (Fig 2b). All comparisons revealed a higher number of down-regulated genes than up-regulated genes. Additionally, Venn diagrams indicated that the DEGs from the off-season vs. normal comparison had very few overlapping genes (<1%) with those from the other two comparisons (depicted in the first and second diagrams in Fig 2c). On the other hand, early vs. normal and late vs. normal comparisons shared approximately 25% of the DEGs (depicted in the third diagram in Fig 2c). These results suggest that the expression pattern of the off-season differed from both the early and late seasons when compared to the normal season.

### Altered spawning seasons affected various biological pathways related to metabolism, cellular processes, and organismal systems

**Fig 3:**
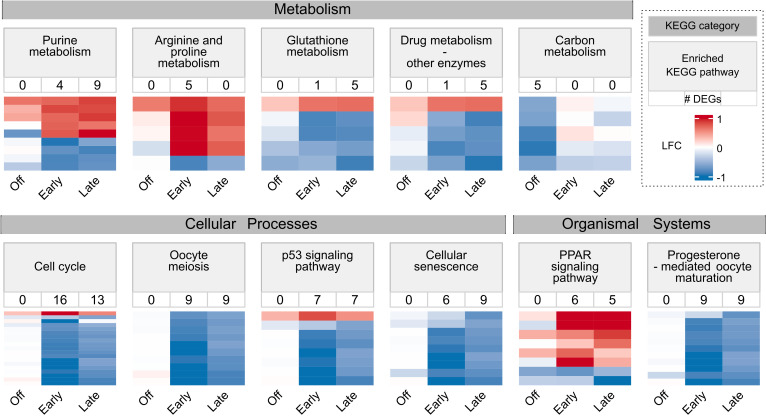
Enriched KEGG pathways for DEGs identified in three pair-wise comparisons. Heatmaps showing log fold changes (LFCs) of genes associated with 11 KEGG pathways enriched through over-representation analysis. LFCs underwent normal shrinkage transformation. Genes were included if identified as DEGs in at least one of the three comparisons: off-season vs normal (Off), early vs normal (Early), and late vs normal (Late). The number below each pathway represents DEG count. KEGG pathways are categorized into metabolism, cellular processes, and organismal systems groups.

To explore the impact of altered spawning seasons on biological pathways, we conducted functional analysis on DEGs using the Kyoto Encyclopedia of Genes and Genomes (KEGG) database. Over-representation analysis (ORA) identified a total of 11 enriched KEGG pathways, which were classified into three functional categories: metabolism, cellular processes, and organismal systems, when comparing the three altered spawning seasons against the normal season (Fig 3).

The off-season vs. normal comparison identified only one enriched pathway, carbon metabolism (Supplementary Table S8 in S1 File). All five DEGs associated with carbon metabolism showed down-regulation (Fig 3).

The early vs. normal comparison revealed six enriched pathways: one in metabolism, three in cellular processes, and two in organismal systems (Supplementary Table S9 in S1 File). Metabolism-related pathways exhibited both up- and down-regulation, while most genes in cellular processes showed significant down-regulation. In organismal systems, PPAR signalling showed up-regulation, while progesterone-mediated oocyte maturation showed down-regulation (Fig 3).

The late vs. normal comparison identified nine enriched pathways: three in metabolism, four in cellular processes, and two in organismal systems (Supplementary Table S10 in S1 File). Remarkably, this comparison shared five enriched pathways with the early vs. normal comparison, indicating similar impacts on biological pathways between them. Moreover, these pathways displayed consistent expression patterns in terms of up- and down-regulation between early and late seasons. Notably, genes associated with cellular processes exhibited strong down-regulation for both seasons (Fig 3).

In summary, the off-season appeared to down-regulate carbon metabolism, while the early and late seasons primarily had strong suppressive effects on pathways associated with cellular processes.

### Altered spawning seasons had an impact on the expression of genes in a similar manner

To further investigate the genes commonly affected by multiple spawning seasons, we used overlapping DEGs (represented as intersections of Venn diagrams in Fig 2c) by applying two filtering steps: selecting DEGs found in at least two comparisons and choosing the top five DEGs from each comparison based on adjusted p-values (Supplementary Table S11 in S1 File). This filtering approach revealed a total of 13 genes (Table 1). Among them, two genes (gene IDs: 106612264 and 106601246) lacked corresponding gene names and symbols. All genes, except *pc* and *pitpnm2*, consistently displayed up- or down-regulation across all spawning seasons, suggesting that all altered spawning seasons had a similar effect on this specific set of genes when compared against the normal season (Table 1).

**Table 1:** List of top five DEGs identified in at least two pairwise comparisons.

Pattern^1^	Gene ID	Symbol	LFC^2^			Gene name^3^	Function
			Off-season	Early	Late		
All Down	106563634	*pcyt1b*	−1.9*	−1.1	−1.6*	choline-phosphate cytidylyltransferase B	Metabolism
	106612672	*bmp5*	−1.2*	−1.2*	−0.7	bone morphogenetic protein 5-like	Signalling
	106585803	*rhobtb4*	−0.3	−1.1*	−0.9*	Rho related BTB domain containing 4	Ubiquitin mediated proteolysis
	106608456	*prox3*	−0.2	−2.3*	−1.7*	prospero homeobox 3	Lymphangiogenesis
	106584736	*tcf12*	−0.7	−3.3*	−2.3*	transcription factor 12	Myogenic regulatory
Up & Down	106592454	*pc*	−1.7*	1.5*	0.9	pyruvate carboxylase, mitochondrial-like	Pyruvate metabolism, TCA cycle, redox
	106568312	*pitpnm2*	0.6	−2.1*	−2.6*	membrane-associated phosphatidylinositol transfer protein 2-like	Cell signalling
All Up	106612264	–	5.2*	4.1	6.1*	Uncharacterized	–
	106601246	–	1.8*	2.2*	1.5	Uncharacterized	–
	106613828	*crim1*	0.3	3.0*	3.0*	cysteine-rich motor neuron 1 protein-like	CNS development, Bone morphogenic and growth factor
	106566973	*krt8*	0.1	1.3*	1.0*	keratin, type II cytoskeletal 8	Liver development
	106583637	*klf15*	2.3	4.5*	3.8*	Krueppel-like factor 15	Metabolism
	106569008	*cyp7a1*	1.4	3.1*	2.9*	cytochrome P450 7A1	Metabolism

^1^All Down: All three comparisons showed down-regulation, Down+Up: Comparisons showed both down- and up-regulation. All Up: All three comparisons showed up-regulation.

^2^Log fold changes of pairwise comparison against the normal season.

* : significant LFC (adjusted p-value < 0.05).

^3^When gene symbols were unavailable, we utilised the gene symbols from either the latest NCBI version or UniProt as substitutes.

These genes can be classified into three categories by relevant biological functions: metabolism (*pcyt1b*, *pc*, *klf15*, and *cyp7a1*) [34,39,40], tissue development (*prox3*, *crim1*, and *krt8*) [41–44], and information processing, which can be further classified into three sub categories as signalling (*bmp5* and *pitpnm2*) [34,45], transcription (*tcf12*) [46], and post-translation (*rhobtb4*) [34]. As these categories are typically associated with growth, all three altered spawning seasons affected a certain set of genes associated with growth regulation in a similar manner, despite PCA plots (Fig 2a) and Venn diagrams (Fig 2c) showing a distinct gene expression pattern of the off-season.

### Overall methylation rates increased until the harvesting stage within and around gene bodies, except near transcription start sites

We conducted DNA methylation analysis on 20 liver samples from larvae across four different spawning seasons (n = 5; detailed statistics in Supplementary Table S12 in S1 File) to investigate the epigenetic effects of altered spawning seasons on DNA methylation.

**Fig 4:**
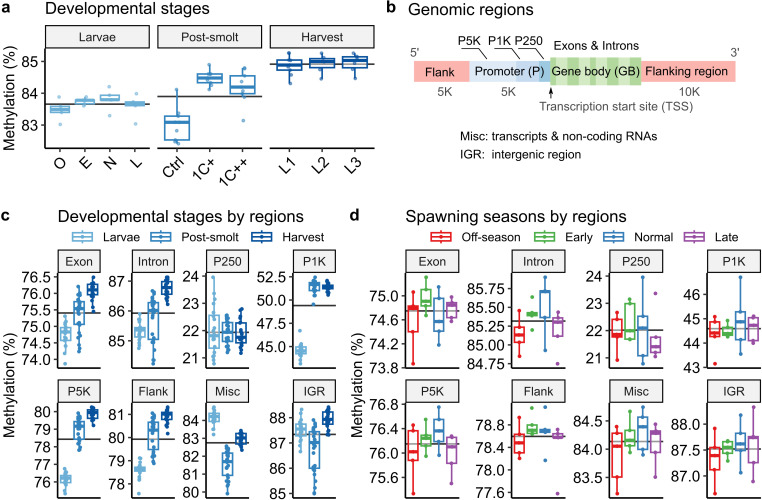
Distribution of methylated CpG sites across genomic regions. Box plots displaying liver DNA methylation status during the lifespan of Atlantic salmon; data from the present study (larvae, light blue) and two other studies: post-smolt (blue) and harvest (dark blue). In the larvae stage, O, E, N, L indicate off-season, early, normal, and late seasons, respectively. While the post-smolt stage contains three groups with different 1C-metabolism nutrient levels (Ctrl, 1C + , and 1C++), the harvest stage contains three groups with different micronutrient levels (L1, L2, and L3) in their diets. Horizontal lines indicate the average rates for each group. **a)** Box plots showing average methylation rates of the samples from three developmental stages. **b)** Genomic region diagram illustrating three regions: flanking regions (Flank, red), promoters (P, blue), and gene bodies (GB, green). The promoter region is divided into three subregions: P250, P1K, and P5K. The gene body includes exons and introns. **c)** Box plots showing average methylation rates of the samples from three developmental stages across eight genomic regions. **d)** Box plots showing average methylation rates of the samples from four spawning seasons across eight genomic regions.

Before analysing the effects of altered spawning seasons, we used RRBS data from two independent studies on liver samples to investigate DNA methylation changes across developmental stages. Specifically, we compared our RRBS data from the larvae stage with the post-smolt stage (approximately 31 weeks) and the harvest stage (around 45 weeks) [19,20]. The post-smolt samples were divided into three groups with different 1C metabolism nutrient levels (Ctrl, 1C + , and 1C++) according to their experimental diets, while the harvest samples were divided into three groups with varying micronutrient levels (L1, L2, and L3) based on their experimental diets. We compared these six groups from post-smolt and harvest developmental stages with our larvae stage samples, which were collected across four spawning seasons (Fig 4a).

Notably, the average methylation rates exhibited a consistent increase from larvae to post-smolt to harvest stages: 83.7%, 83.9%, and 84.9% for larvae, post-smolt, and harvest stages, respectively (Kruskal-Wallis, p-value <  0.001, black horizontal lines in Fig 4a). Within the larvae group, the average rate of the normal group was higher than that of the off-season group, but the difference was not significant. Within the post-smolt group, the Ctrl group showed a significantly lower methylation rate than the other two groups (pair-wise Wilcoxon test with the Holm correction, p-values <  0.05; Supplementary Table S13 in S1 File). The Ctrl group was given lower methionine along with B-vitamins as the 1C metabolism nutrients compared to other groups (1C + and 1C++) [20].

To examine regional DNA methylation patterns, CpG sites were categorised into three genomic regions based on their positions relative to nearby genes: gene body (GB; including exons and introns), promoter (P; including P250, P1K, and P5K), flanking regions, miscellaneous regions (Mics, including various transcripts and non-coding RNA), and intergenic regions (IGR) (Fig 4b; see Methods and Materials for definitions).

The trend of the rate increment became more noticeable when the rates were examined separately by different genomic regions (Fig 4c). In most regions, including gene bodies, promoter regions except for P250, and flanking regions, the samples from the harvest stage exhibited significantly higher average rates, followed by the post-smolt samples, and then the larvae samples (pair-wise Wilcoxon test with the Holm correction, p-values <  0.05; Supplementary Table S14 in S1 File). Specifically, within the two promoter regions (P1K and P5K), both the post-smolt and harvest stages showed a significant increase in methylation rates compared to the larvae stage. Moreover, methylation rates were generally lower in P250 (~22%) and P1K (~49%) compared to other regions (75% ~  88%).

This comparative investigation suggests a potential increase in methylation rates as salmon progress in growth, at least until the harvesting stage, while also noting that methylation rates near transcription start sites (TSSs) tend to remain lower and more stable than in other genomic regions.

### The off-season showed non-significant but consistently lower methylation rates compared to the normal and early seasons

To investigate the regional shift of the methylation rates among four spawning seasons, we applied the same methods used for the comparison analysis between developmental stages. Unlike the result of the developmental stages, spawning seasons exhibited no significant differences in methylation rates when separated by genomic regions (Fig 4d; pair-wise Wilcoxon test with the Holm correction in Supplementary Table S15 in S1 File). Despite the non-significant differences, the average rates of the off-season samples were lower than those of the normal and early seasons in all regions (Supplementary Table S16 in S1 File). Nonetheless, the average rates of the off-season were higher than those of the late season in the two promoter regions (P250 and P5K) and flanking regions.

### Differential methylation patterns indicated higher dynamic regulation in promoter regions compared to other regions

**Fig 5:**
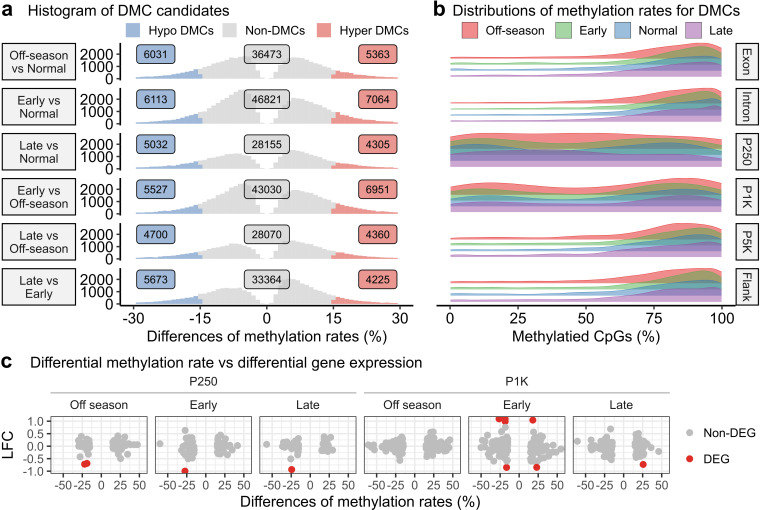
DNA methylation differences and correlations with gene expression. **a)** Histograms displaying distributions of methylation rate differences in six pairwise comparisons. Hypo-methylated DMCs in blue, hyper-methylated DMCs in red, non-DMCs in grey. All CpG sites in plots showed significant methylation differences. **b)** Ridge density plots illustrating distribution patterns of methylated CpG sites across four spawning seasons: off-season (red), early (green), normal (blue), and late (purple). CpG sites were selected if at least one site identified as DMC in any of six comparisons. **c)** Scatter plots showing the relationship between methylation rate differences and log fold changes (LFCs) of gene expression. DMCs were selected from three comparisons: off-season vs. normal (Off-season), early vs. normal (Early), and late vs. normal (Late), and also selected based on two regions, P250 and P1K. Corresponding genes were linked to these DMCs chosen, and their LFCs were transformed by normal shrinkage. Data points are color-coded to indicate DEGs in red and non-DEGs in grey.

We conducted six pairwise comparisons to identify CpG sites with differential methylation (Fig 5a). Unlike the analysis of the RNA-seq data, we considered all six possible pair-wise comparisons to capture a comprehensive pattern of methylation differences with our RRBS data. Remarkably, all comparisons consistently demonstrated similar characteristics, for both the number of differentially methylated CpGs (DMCs) and the balanced ratio of hypo- and hyper-methylation (Fig 5a).

Further investigation focused on a set of 10,308 common DMCs, identified in at least one comparison. Distributions of methylation rates for these common DMCs exhibited a distinct left-skewed pattern across the exon, intron, P5K, and flanking regions (Fig 5b). This observation strongly suggested that methylation differences resulting from altered spawning seasons primarily occurred within regions that were already highly methylated. In contrast, the P250 and P1K regions displayed nearly uniform methylation rates across a range of 0–100%, indicating significant methylation differences occurred irrespective of the underlying methylation rates. These findings imply a potentially greater level of dynamic regulation of DNA methylation in P250 and P1K compared to the other regions.

### Filtering multiple DMCs identified key genes affected by spawning season alterations

We examined genes with multiple DMCs in three pairwise comparisons against the normal season. Since most genes had only a single DMC, especially in promoters (~75% in P250 and ~ 70% in P1K; Supplementary Figure S1 in S1 File), we avoided identifying differentially methylated regions (DMRs) using a sliding window approach. Instead, we filtered out genes strongly supported by multiple DMCs based on specific criteria (see Methods and Materials), resulting in a total of 11 genes with multiple DMCs on their promoters (Table 2; see the details in Supplementary Table S17 in S1 File).

**Table 2:** List of genes with multiple DMCs in their promoter region.

Pattern^1^	Gene ID	Symbol	DMC^2^			Gene name	Function
			Off-season	Early	Late		
All hypo	106563027	*slc31a1*	Hypo (3)*	–	Hypo (3)*	solute carrier family 31 member 1	Transporter
Hyper & Hypo	106567512	*stk32a*	–	Hypo (2)	Hypo (2), Hyper (1)*	serine/threonine-protein kinase 32A	Signalling
	106607889	*inf2*	Hypo (1)	Hyper (3)*	–	inverted formin-2-like	Actin filament polymerization
	106584059	*scp-2*	Hyper (4)*	Hypo (1)	Hyper (1)	sterol carrier protein 2-like	Lipid transfer, transporter
	106579168	*klhl11*	Hypo (1), Hyper (2)*	–	–	kelch-like protein 11	Ubiquitination
All hyper	100196480	*ctl2b*	Hyper (3)	Hyper (7)*	Hyper (2)	CTLA-2-beta	Unknown
	106573279	–	–	Hyper (1)	Hyper (3)*	Uncharacterized	–
	106583651	*helz2*	Hyper (4)*	–	–	helicase with zinc finger domain 2-like	Transcriptional coactivator
	106581656	*lrrfip2*	Hyper (3)*	–	–	leucine-rich repeat flightless-interacting protein 2-like	Signalling
	106609256	*prrx2*	Hyper (3)*	–	–	paired mesoderm homeobox protein 2-like	Transcription
	106576160	*ttll12*	–	Hyper (3)*	–	tubulin tyrosine ligase-like family, member 12	Post-translational modification

^1^All hypo: All three comparisons showed hypo-methylated DMCs, Hyper & Hypo: Comparisons showed both hypo- and hyper-methylated DMCs. All hyper: All three comparisons showed hyper-methylated DMCs.

^2^Either hyper or hypo-methylated DMC against the normal season. The number in brackets represents the number of DMCs.

*: genes selected by filtering of multiple DMCs.

All 11 genes were identified by a single comparison except for *slc31a1*, which was identified in two comparisons (off-season vs. normal and late vs. normal). Most genes (9 out of 11) exhibited either hypo-methylated or hyper-methylated DMCs, but not both, within a single comparison (Table 2). Furthermore, approximately 60% of the genes (7 out of 11) consistently displayed either hypo-methylation or hyper-methylation even across multiple comparisons (‘All hypo’ and ‘All hyper’ in the first column named ´Pattern´ of Table 2, respectively).

Literature analysis associated these genes with two biological functions: development (*inf2*) and information processing, which can be further classified into four sub categories as transporter (*slc31a1* and *scp-2*) [47,48], signalling (*stk32a* and *lrrfip2*) [26,49], post-translation (*klhl11* and *ttll12*) [26,50], and transcription (*helz2* and *prrx2*) [51,52]. However, *ctl2b* and an uncharacterised gene (Gene ID: 106573279) had no clear associations with specific biological functions. Although the DMCs on these genes were potentially linked to the underlying gene expression, none of the 11 genes were identified as DEGs in our differential gene expression analysis.

### Altered spawning seasons influenced gene expression and DNA methylation of cell cycle-related genes

To investigate potential associations between DNA methylation and gene expression, we combined DEGs and DMCs from three pairwise comparisons against the normal season (off-season vs. normal, early vs. normal, and late vs. normal) in P250 and P1K. This resulted in a total of seven DEGs that contain a total of 11 DMCs in their promoters (Fig 5c, Table 3, Supplementary Table S18 in S1 File).

**Table 3:** List of DEGs that contain DMCs in their promoter region.

Gene ID	Symbol	DMC^1^			DEG^2^			Gene name	Function
		Off-season	Early	Late	Off-season	Early	Late		
106562317	*caprin-1*	Hypo	(Hypo)	(Hypo)	Down	(Down)	(Down)	caprin-1	Cell cycle
106599887	*cyp8b1*	Hypo	–	–	Down	–	–	5-beta-cholestane-3-alpha,7-alpha-diol 12-alpha-hydroxylase	Metabolism, cytochrome P450
106588407	*kifc1*	–	Hypo	Hypo	–	Down	Down	carboxy-terminal kinesin 2	Cell cycle, Meiosis
106582038	*adrenodoxin*	–	Hypo		–	Down	–	adrenodoxin	Metabolism, cytochrome P450
106604665	*slc43a1a*	–	Hypo	–	–	Up	(Up)	solute carrier family 43 member 1a	Transporter
106561604	*aurkb*	(Hyper)	Hyper	Hyper	–	Down	Down	aurora kinase B	Cell cycle
106570052	*lpin1*	–	Hyper	–	–	Up	–	phosphatidate phosphatase LPIN1	Metabolism

^1^Either hypo- or hyper-methylated DMC against the normal season. Parentheses indicate that the site was identified as DMC, but the corresponding genes were not identified as stringent DEG. ^2^Either up- or down-regulation against the normal season. Parentheses indicate that down- or up-regulation was determined by non-stringent DEGs.

In addition to the significant results, we expanded the list of seven DEGs with additional information from all pair-wise comparisons (additional entries were marked with parentheses in Table 3). Among the identified genes, three (*caprin-1*, *kifc1*, and *aurkb*) exhibited consistent regulations of DMCs and DEGs compared to the normal season. Specifically, *caprin-1* and *kifc1* had hypo-methylated CpG sites, while *aurkb* had hyper-methylated CpG sites; all three genes showed down-regulated gene expression (Table 3). Notably, these three genes (*caprin-1*, *kifc1*, and *aurkb*) were associated with cell cycle regulation [53–55], while the remaining genes were linked to metabolism (*cyp8b1*, *adrenodoxin*, and *lpin1*) [34,56,57] and transport (*slc43a1a*) [58]. These findings indicate that altered spawning seasons may have affected gene expression and epigenetic regulation of several genes associated with cell cycle control.

## Discussion

In the present study, we investigated the influence of altered spawning seasons on gene expression and DNA methylation in offspring. Previous studies emphasized the significant impacts of these changes on the nutritional status of both broodstock and their offspring, with no observed effects on the body weights of broodstock but in offspring [7,8]. Our primary aim was to reveal the transcriptomic and epigenetic effects resulting from observed variations by comparing liver samples from larvae at the first feeding stage.

### What insights do our omics analyses provide?

Gene expression analysis revealed distinct patterns associated with altered spawning seasons. Specifically, early and late seasons exhibited similar gene expression patterns when compared to the normal season. The differential expression of genes associated with metabolism, cellular processes, and organismal systems indicates that spawning seasons can have wide-ranging effects on biological pathways related to growth and development. Furthermore, the analysis with overlapped DEGs revealed that all three altered spawning seasons similarly affected genes associated with metabolism, tissue development, and information processing. Hence, while early and late seasons influenced a wide range of biological pathways in a similar manner, all three seasons, including the off-season, also similarly affected a certain set of genes, especially those strongly differentiated from the normal season.

DNA methylation analysis provided valuable insights into the epigenetic effects of different spawning seasons. Altered spawning seasons led to increased methylation rates for the early and normal seasons. Notably, these shifts primarily occurred in regions that were already highly methylated, but the rates were altered independently of the underlying methylation rates in promoter regions around transcription start sites. In addition, our findings across different developmental stages imply that methylation rates could serve as an indicator of age. However, more research is needed to understand the mechanism of methylation rate shifting and potential applications in aquaculture as well as age measure for stock assessment.

Several genes with multiple DMCs exhibited consistent hypo- or hyper-methylation patterns across multiple spawning seasons in promoter regions, suggesting a common epigenetic response to altered spawning seasons. These genes were associated with key biological functions, such as development and information processing, potentially indicating their role in growth performance. Although both gene expression and DNA methylation analyses resulted in similar biological functions impacted by altered spawning seasons, they showed little overlap between DEGs and genes with DMCs. This could be attributed to regulatory differences between gene expression and DNA methylation within the same biological pathway, or it might be a limitation caused by the restriction enzyme used in RRBS.

The observed correlations between DNA methylation and gene expression in our liver samples were weaker than anticipated. Note that the measurements were taken from siblings rather than the same individual due to the limited size of larvae livers. Notably, our findings also revealed inconsistent correspondence between hypo-methylation and active gene expression as well as between hyper-methylation and gene suppression in the liver tissue analysed. These results suggest that the regulation of DNA methylation is more intricate than commonly assumed as it is known that high promoter CpG methylation reduces gene expression, while low promoter CpG methylation allows for active gene expression [59]. Within promoter regions, not only DNA methylation but several other factors, including underlying gene expression levels, transcription factor binding sites, and chromatin remodelling, likely play a role in regulating actual gene expression.

### What are the significant biological differences observed in offspring?

Off-season vs. normal: Off-season spawning in June, facilitated through a RAS, resulted in Atlantic salmon offspring with lower larval weights compared to the normal and late seasons [7,8]. Off-season eggs also exhibited reduced levels of vitamin B12 and lipid classes relative to normal and late seasons [7,8]. Gene expression analysis indicated significant down-regulation of genes associated with central carbon metabolism (CCM). CCM involves several biological pathways, including the citric acid cycle, and acts as a major source of energy for growth and development. Moreover, lipids are interconnected with CCM through the utilisation of acetyl-CoA as a central metabolite [60]. Among the six genes with multiple DMCs from the off-season vs. normal comparison, sterol carrier protein2-like (*scp-2*) contained four hyper-methylated DMCs in its promoter. The *scp-2* gene encodes a transfer protein that plays a key role in intracellular lipid transport [48]. Overall, RAS-based off-season spawning appeared to impact lipid-mediated regulations both transcriptionally and epigenetically. This negative impact on lipid-related mechanisms potentially accounted for impaired growth performance observed in body weight as our previous research indicated the importance of gene expression and epigenetic regulation of lipid regulation, specifically involving acetyl-CoA, for growth performance [19].

Early season vs. normal season: Offspring resulting from early season spawning in September also exhibited lower weights at the larval stage compared to those from normal and late seasons. However, the nutritional status of offspring eggs displayed higher levels of SAM/SAH, lysine, glutamine, and alanine [7,8]. Gene expression analysis revealed significant down-regulation of genes associated with cell cycle regulation, while genes involved in metabolism showed both down- and up-regulation. Among the five genes that exhibited differential expression and had at least one DMC in their promoters, two were associated with cell-cycle regulation. Specifically, the carboxy-terminal kinesin 2 (*kifc1*) gene promotes mitotic spindle assembly [54], and the aurora kinase B (*aurkb*) gene is involved in mitotic progression [55]. The *kifc1* gene was hypomethylated, while the *aurkb* gene was hypermethylated. Consequently, the early spawning season had an impact on cell-cycle regulation both transcriptionally and epigenetically. However, it is challenging to hypothesise that this negative impact on cell-cycle regulation was linked to impaired growth performance because similar effects were observed for the late season.

Late season vs. normal season: Late season spawning in January showed that offspring weights were almost the same, but slightly heavier than those from the normal season. The nutritional status of offspring eggs also exhibited similar levels of various nutrients and metabolites to the normal season, except for higher vitamin B12 and lower B-alanine [8]. Gene expression patterns in the late season were comparable to those observed in the early season, showing significant down-regulation of genes related to cellular processes, especially cell cycle regulation. Two genes, *kifc1* and *aurkb*, showed differential expression and had at least one DMC in their promoters, and both were associated with cell-cycle regulation. Hence, the late season showed similar growth performance and nutritional status to the normal season, while the gene expression pattern was similar to the early season.

Three altered spawning seasons vs. normal season: Although strong similarities were observed in gene expression patterns between early and late spawning seasons, certain genes exhibited common regulation in both gene expression and DNA methylation across all three altered spawning seasons. Among the four DEGs identified in the off-season, choline-phosphate cytidylyltransferase B (*pcyt1b*) and bone morphogenetic protein 5-like (*bmp5*) showed concordant down-regulation across all three altered spawning seasons. The *pcyt1b* gene encodes an enzyme involved in phosphatidylcholine biosynthesis, playing essential roles in multiple metabolic pathways [26], while the *bmp5* gene is involved in signalling [34]. Additionally, all DMCs found in the promoter of the cytotoxic T lymphocyte-associated protein 2 beta (*ctl2b*) gene were hyper-methylated across all three altered spawning seasons. The function of *ctl2b* remains unknown, but it shares similarities with genes encoding cysteine proteinase, an enzyme involved in protein breakdown within cells [61]. Furthermore, among genes that were both differentially expressed and had at least one DMC in their promoters, the *caprin-1* gene exhibited consistent regulation, with all DMCs being hypo-methylated and its expression being suppressed. The *caprin-1* gene is associated with cell cycle regulation, playing a crucial role in cellular activation or proliferation [53]. This indicates that the off-season also influenced cell cycle regulation to a certain degree.

### What factors contributed to our results?

Temperature and light are the primary factors influencing the spawning season although other variables, such as the duration of maturation, may also play a role. The present study was designed to explore general influences rather than isolate the factor with the most significant impact on our findings. A recent study also showed that altered spawning seasons negatively affected egg quality, though it was structured to observe broader effects rather than identify specific causal factors [5]. A specialised study design would be essential to separate the influences of temperature and light. For example, recent research on Atlantic salmon has examined the effects of variations in light and temperature on redox regulation [62,63]. A new study using similar conditions but spanning multiple generations would provide valuable insight into the actual causal factors in the field of intergenerational epigenetics.

### Why are our findings important in the context of aquaculture and nutritional studies?

Our study unveiled the significant effects of altered spawning season on offspring gene expression and DNA methylation patterns in Atlantic salmon. These findings provide valuable insights into assessing current and future growth potential, which can be challenging to determine using only nutritional status and growth performance measures. Understanding the gene regulation and epigenetic responses to altered spawning seasons will help develop strategies to optimize growth and production in aquaculture practices. Additionally, our study can be linked to the potential impact of increasing global ocean temperatures on the spawning of aquatic vertebrates, as well as the northward shift in habitats followed by changes in photoperiod, as both temperature and light are key abiotic factors influencing spawning seasons. Even though genetic and epigenetic profiles can be species- and even tissue-specific, the approaches we demonstrated here could be applied to other fish species whose spawning seasons are artificially altered in aquaculture practices. Nonetheless, further studies are needed to elucidate the fundamental effects caused by spawning season alterations across various species.

## Conclusions

The present study emphasises the substantial impact of altering broodstock spawning seasons on gene expression and DNA methylation patterns in Atlantic salmon offspring. Our observations indicate that RAS-based off-season spawning exerts an influence on lipid-mediated regulations in offspring, while sea-pen based early and late seasons affect the regulation of the offspring’s cellular processes, especially cell cycle regulation. These effects potentially play a crucial role in shaping both current and future growth performance.

These findings have important implications for aquaculture and nutritional studies, as they provide valuable tools for assessing growth potential and optimizing production strategies. Future research focusing on tissue and developmental stage-specific DNA methylation maps will enhance the accuracy of growth performance estimation and provide further insights into the gene regulation and epigenetic responses to altered spawning conditions.

## Supporting information

S1 File Altered spawning seasons of Atlantic salmon broodstock genetically and epigenetically influence cell cycle and lipid-mediated regulations in their offspring. (PDF)
