# Supplementary material for: Altered spawning seasons of Atlantic salmon broodstock transcriptionally and epigenetically influence cell cycle and lipid-mediated regulations in their offspring
Source: PLoS One. 2025 Feb 24;20(2):e0317770. doi: 10.1371/journal.pone.0317770 (PMC11849821; doi:10.1371/journal.pone.0317770)
Supplement: S1 File — (PDF) [file pone.0317770.s001.pdf]

S1 Info: Supplementary information

Altered spawning seasons of Atlantic salmon broodstock genetically and epigenetically influence cell cycle and lipid-mediated regulations in their offspring

Takaya Saito, Marit Espe, Maren Mommens, Christoph Bock, Jorge M.O. Fernandes, Kaja H. Skjaerven

Tables

Supplementary Table 1. Feeding regime overview for four spawning groups.

	Off-season	Early	Normal	Late
**Starvation start date**	03.03.2017	16.04.2017	26.07.2017	26.07.2017
**Stripping date**	20.06.2017	26.09.2017	23.11.2017	08.01.2018
**Days of starvation**	109	163	120	166
**Egg/liter**	6155	5835	5082	4613
**Day° newly fertilized egg**	2	2	2	2
**Day° eye staged egg**	364	363	362	359
**Day° start feeding larvae**	994	991	979	981

Supplementary Table 2. Weights of broodstock and off-spring larvae in four spawning seasons.

Stage	Unit	Season	Weight mean	Weight SD	Tukey's HSD[1]
Broodstock	kg	Off-season	12.10	1.55	-
		Early	11.52	1.02	-
		Normal	11.74	0.84	-
		Late	12.56	0.85	-
Offspring	g	Off-season	154.12	14.55	a
		Early	142.04	15.52	b
		Normal	184.37	20.42	c
		Late	191.68	20.76	d

[1]Letters represent Tukey's HSD compact letter display after ANOVA tests ($p<0.05$).

Supplementary Table 3. Nutritional status of the 1C metabolism in eggs.

Nutrient	Unit	Season	Mean	SD	Tukey's HSD[1]
**Vit B6**	mg/kg ww	Off-season	1.74	0.25	-
		Early	1.56	0.05	-
		Normal	1.76	0.18	-
		Late	1.81	0.19	-
**Folate**	mg/kg ww	Off-season	1.64	0.12	ab
		Early	1.26	0.14	a
		Normal	1.82	0.25	b
		Late	1.60	0.25	ab
**Vit B12**	µg/100 g ww	Off-season	62.84	10.94	a
		Early	108.34	7.32	b
		Normal	107.49	19.20	b
		Late	166.76	8.25	c
**SAM/SAH**	-	Off-season	3.70	0.49	a
		Early	4.72	0.29	b
		Normal	3.17	0.36	ac
		Late	2.71	0.34	c

[1]Letters represent Tukey's HSD compact letter display after ANOVA tests ($p<0.05$).

Supplementary Table 4. Nutritional status of lipid classes in eggs.

Nutrient	Unit	Season	Mean	SD	Tukey's HSD[1]
**Cholesterol**	mg/g ww	Off-season	3.20	0.67	a
		Early	5.76	0.59	b
		Normal	5.80	0.22	b
		Late	5.78	0.08	b
**Sum of lipids**	mg/g ww	Off-season	97.52	17.33	a
		Early	167.80	8.87	b
		Normal	173.40	14.81	b
		Late	167.60	9.66	b

[1]Letters represent Tukey's HSD compact letter display after ANOVA tests ($p<0.05$).

Supplementary Table 5. Nutritional status of the citric acid cycle in eggs.

Nutrient	Unit	Season	Mean	SD	Tukey's HSD[1]
**Glutamate**	µmol/g ww	Off-season	3.44	0.69	a
		Early	4.08	0.30	a
		Normal	1.87	0.43	b
		Late	2.15	0.51	b
**L-Lysine**	µmol/g ww	Off-season	0.62	0.17	a
		Early	0.89	0.10	b
		Normal	0.53	0.10	a
		Late	0.49	0.03	a

[1]Letters represent Tukey's HSD compact letter display after ANOVA tests ($p<0.05$).

Supplementary Table 6. Nutritional status of the Cahill cycle in eggs.

Nutrient	Unit	Season	Mean	SD	Tukey's HSD[1]
**L-Alanine**	µmol/g ww	Off-season	1.53	0.47	a
		Early	2.05	0.20	b
		Normal	1.39	0.14	a
		Late	1.57	0.06	ab
**L-Glutamine**	µmol/g ww	Off-season	1.16	0.26	a
		Early	1.61	0.11	b
		Normal	0.79	0.06	c
		Late	0.87	0.12	c
**Urea**	µmol/g ww	Off-season	0.21	0.08	a
		Early	0.28	0.08	ab
		Normal	0.38	0.11	bc
		Late	0.50	0.09	c
**B-Alanine**	µmol/g ww	Off-season	0.05	0.01	ab
		Early	0.10	0.03	c
		Normal	0.07	0.01	b
		Late	0.03	0.01	a

[1]Letters represent Tukey's HSD compact letter display after ANOVA tests ($p<0.05$).

Supplementary Table 7. List of RNA-seq samples with alignment and mapping statistics.

Season	Sample	M Seqs[1]	M Aligned[2]	% Aligned	M Assigned[3]	% Assigned
**Off-season**	O1	44.5	37.4	84.0%	33.1	61.3%
	O2	46.4	38.8	83.7%	33.8	60.0%
	O3	46.2	38.6	83.5%	33.6	60.0%
	O4	46.6	39.2	84.1%	34.4	61.3%
	O5	35.6	29.8	83.8%	26.7	61.6%
**Early**	E1	44.5	37.7	84.8%	32.9	61.6%
	E2	42.9	36.4	84.8%	32.1	62.6%
	E3	41.1	34.7	84.3%	30.8	62.7%
	E4	36.3	30.5	83.8%	27.3	62.2%
	E5	36.2	30.6	84.5%	27	61.4%
**Normal**	N1	33.2	27.8	83.7%	25	62.0%
	N2	43.7	36.4	83.2%	32.8	63.4%
	N3	44	36.9	84.0%	32.9	62.0%
	N4	39.8	33.6	84.6%	29.7	62.0%
	N5	37.1	31.3	84.4%	28.2	62.9%
**Late**	L1	37.2	31.5	84.9%	27.5	62.0%
	L2	47.4	39.9	84.2%	35.7	62.4%
	L3	41.9	35.3	84.2%	31.3	62.1%
	L4	43.3	36.5	84.3%	32.5	62.1%
	L5	44.4	37.5	84.5%	33.1	61.9%

[1]Number of reads in millions. [2]Number of uniquely aligned reads to the reference genome. [3]Number of uniquely mapped reads to genes for quantification.

Supplementary Table 8. One enriched KEGG pathway identified by ORA for off-season vs. normal DEGs.

ID	Enriched KEGG pathway	GeneRatio	BgRatio	P-value	Adjusted p-value
**sasa01200**	Carbon metabolism	5/44	247/11637	2.29E-03	4.58E-02

Supplementary Table 9. Six enriched KEGG pathways identified by ORA for early vs. normal DEGs.

ID	Enriched KEGG pathway	GeneRatio	BgRatio	P-value	Adjusted p-value
**sasa04110**	Cell cycle	16/104	275/11546	2.81E-09	2.39E-07
**sasa04914**	Progesterone-mediated oocyte maturation	9/104	204/11546	9.07E-05	3.86E-03
**sasa04115**	p53 signaling pathway	7/104	147/11546	3.56E-04	9.63E-03
**sasa04114**	Oocyte meiosis	9/104	253/11546	4.53E-04	9.63E-03
**sasa03320**	PPAR signaling pathway	6/104	137/11546	1.47E-03	2.49E-02
**sasa00330**	Arginine and proline metabolism	5/104	99/11546	1.97E-03	2.79E-02

Supplementary Table 10. Nine enriched KEGG pathways by ORA for late vs. normal DEGs.

ID	Enriched KEGG pathway	GeneRatio	BgRatio	P-value	Adjusted p-value
**sasa04110**	Cell cycle	13/91	278/11587	2.38E-07	2.14E-05
**sasa04914**	Progesterone-mediated oocyte maturation	9/91	204/11587	3.07E-05	1.38E-03
**sasa04115**	p53 signaling pathway	7/91	148/11587	1.59E-04	3.63E-03
**sasa04114**	Oocyte meiosis	9/91	253/11587	1.61E-04	3.63E-03
**sasa00230**	Purine metabolism	9/91	269/11587	2.55E-04	4.59E-03
**sasa00480**	Glutathione metabolism	5/91	90/11587	6.95E-04	1.04E-02
**sasa00983**	Drug metabolism - other enzymes	5/91	102/11587	1.22E-03	1.57E-02
**sasa04218**	Cellular senescence	9/91	375/11587	2.67E-03	2.41E-02
**sasa03320**	PPAR signaling pathway	5/91	137/11587	4.43E-03	3.63E-02

Supplementary Table 11. Top 5 DEGs identified at least two pairwise comparisons.

Comp[1]	Gene ID	LFC	Adjusted[2]	Symbol[3]	Gene name
**Off-season**	106612264	5.22	6.04E-04	-	-
	106563634	-1.87	3.54E-03	pcyt1b	choline-phosphate cytidylyltransferase B
	106601246	1.85	5.52E-03	(cbx7)	(Chromobox 7)
	106612672	-1.20	1.34E-02	bmp5	bone morphogenetic protein 5-like
	106592454	-1.70	3.59E-02	pc	pyruvate carboxylase, mitochondrial-like
**Early**	106608456	-2.28	9.69E-16	prox3	prospero homeobox 3
	106613828	3.01	9.27E-15	crim1	cysteine-rich motor neuron 1 protein-like
	106566973	1.27	2.83E-09	krt8	keratin, type II cytoskeletal 8
	106585803	-1.12	1.39E-08	rhobtb4	Rho related BTB domain containing 4
	106584736	-3.34	1.14E-07	tcf12	transcription factor 12
**Late**	106583637	3.85	6.75E-17	klf15	Krueppel-like factor 15
	106608456	-1.74	3.44E-09	prox3	prospero homeobox 3
	106568312	-2.63	4.18E-09	pitpnm2	membrane-associated phosphatidylinositol transfer protein 2-like
	106613828	3.02	1.32E-08	crim1	cysteine-rich motor neuron 1 protein-like
	106569008	2.93	1.36E-07	cyp7a1	cytochrome P450 7A1

[1]Pair-wise comparisons against the normal season. [2]Adjusted p-value. [3]When gene symbols were unavailable, we utilised the gene symbols from either the latest NCBI version or UniProt as substitutes. The gene symbol of 106601246 (cbx7) was estimated by OrthoDB.

Supplementary Table 12. List of RRBS samples with alignment and methylation calling statistics.

Season	Sample	M Seqs[1]	% Aligned[2]	% mCpG[3]	% mCHG[4]	% mCHH[5]	M C's[6]
**Off-season**	O1	56.4	49.3%	80.6%	1.0%	0.9%	275.8
	O2	72.1	49.4%	80.9%	1.0%	0.9%	353.1
	O3	45.6	49.1%	80.9%	1.0%	0.9%	220.3
	O4	57.1	49.3%	80.5%	1.0%	0.9%	276
	O5	49.2	48.6%	80.9%	1.1%	1.0%	230.2
**Early**	E1	65.2	46.4%	82.0%	1.0%	0.9%	300
	E2	73.3	48.9%	81.1%	1.0%	0.9%	348.1
	E3	59.8	49.5%	80.8%	1.0%	0.9%	294.7
	E4	45.6	49.2%	80.4%	1.0%	0.9%	217.6
	E5	53	50.0%	80.0%	1.0%	0.9%	262.2
**Normal**	R1	51	50.9%	79.7%	1.0%	0.9%	254.5
	R2	50.1	50.4%	80.8%	1.0%	0.9%	246.6
	R3	60.6	49.0%	80.4%	1.0%	0.9%	291.6
	R4	49.4	49.2%	80.9%	1.0%	0.9%	240.4
	R5	55	49.5%	81.6%	1.0%	0.9%	265.5
**Late**	L1	42.3	49.0%	80.6%	1.0%	0.9%	202.7
	L2	57.9	48.6%	81.1%	1.1%	1.0%	274.1
	L3	60	48.5%	80.8%	1.0%	0.9%	286.1
	L4	44	48.9%	80.4%	1.0%	0.9%	210.6
	L5	43.5	49.0%	81.2%	1.0%	0.9%	213

[1]Number of reads in millions. [2]Number of uniquely aligned reads to the reference genome. [3,4,5]Percentage of methylated cytosine in CpG, CHG, and CHH sites, respectively where H represents non-cytosine. [6]Count of total CpG sites with methylation calling information in millions.

Supplementary Table 13. Comparisons of methylation raters between treatment groups in three life stages.

Stage	Group1	Group2	Adjusted p-value[1]	Significant[2]
**Larvae**	Early	Off-season	0.37	
	Normal	Off-season	0.37	
	Late	Off-season	0.37	
	Normal	Early	0.37	
	Late	Early	0.55	
	Late	Normal	0.37	
**Post-smolt**	1C+	Ctrl	2.47E-04	***
	1C++	Ctrl	1.85E-03	*
	1C++	1C+	0.19	
**Harvest**	L2	L1	0.95	
	L3	L1	0.95	
	L3	L2	0.95	

[1]P-values were adjusted by Holm. [2]Distributions were significantly different ('*': adjusted p-value<0.05, '**': adjusted p-value<0.01, "***': adjusted p-value<0.001).

Supplementary Table 14. Comparisons of methylation raters between life stages by genomic regions.

Region	Group1	Group2	Adjusted p-value[1]	Significant[2]
**Exon**	Post-smolt	Larvae	1.07E-04	***
	Harvest	Larvae	2.23E-11	***
	Harvest	Post-smolt	4.15E-07	***
**Intron**	Post-smolt	Larvae	1.97E-02	*
	Harvest	Larvae	2.23E-11	***
	Harvest	Post-smolt	3.82E-09	***
**P250**	Post-smolt	Larvae	0.92	
	Harvest	Larvae	0.92	
	Harvest	Post-smolt	0.92	
**P1K**	Post-smolt	Larvae	6.15E-13	***
	Harvest	Larvae	1.11E-11	***
	Harvest	Post-smolt	0.26	
**P5K**	Post-smolt	Larvae	6.15E-13	***
	Harvest	Larvae	1.11E-11	***
	Harvest	Post-smolt	9.05E-08	***
**Flank**	Post-smolt	Larvae	1.38E-11	***
	Harvest	Larvae	1.38E-11	***
	Harvest	Post-smolt	2.69E-06	***
**Misc**	Post-smolt	Larvae	6.15E-13	***
	Harvest	Larvae	2.23E-10	***
	Harvest	Post-smolt	4.03E-12	***
**IGR**	Post-smolt	Larvae	1.56E-03	**
	Harvest	Larvae	1.56E-03	**
	Harvest	Post-smolt	8.08E-08	***

[1]P-values were adjusted by Holm. [2]Distributions were significantly different ('*': adjusted p-value<0.05, '**': adjusted p-value<0.01, "***': adjusted p-value<0.001).

Supplementary Table 15. Comparisons of methylation raters between spawning seasons by genomic regions.

Region	Group1	Group2	Adjusted p-value[1]	Significant[2]
**Exon**	Early	Off-season	0.62	
	Normal	Off-season	0.84	
	Late	Off-season	0.66	
	Normal	Early	0.62	
	Late	Early	0.62	
	Late	Normal	0.66	
**Intron**	Early	Off-season	0.30	
	Normal	Off-season	0.30	
	Late	Off-season	0.84	
	Normal	Early	0.66	
	Late	Early	0.33	
	Late	Normal	0.30	
**P250**	Early	Off-season	1	
	Normal	Off-season	1	
	Late	Off-season	1	
	Normal	Early	1	
	Late	Early	1	
	Late	Normal	1	
**P1K**	Early	Off-season	1	
	Normal	Off-season	1	
	Late	Off-season	1	
	Normal	Early	1	
	Late	Early	1	
	Late	Normal	1	
**P5K**	Early	Off-season	0.66	
	Normal	Off-season	0.62	
	Late	Off-season	0.84	
	Normal	Early	0.63	
	Late	Early	0.62	
	Late	Normal	0.62	
**Flank**	Early	Off-season	0.29	
	Normal	Off-season	0.63	
	Late	Off-season	1	
	Normal	Early	1	
	Late	Early	0.29	
	Late	Normal	0.62	
**Misc**	Early	Off-season	0.84	
	Normal	Off-season	0.84	
	Late	Off-season	0.84	
	Normal	Early	0.84	
	Late	Early	0.84	
	Late	Normal	0.84	
**IGR**	Early	Off-season	0.83	
	Normal	Off-season	0.83	
	Late	Off-season	0.83	
	Normal	Early	0.83	
	Late	Early	0.83	
	Late	Normal	1	

[1]P-values were adjusted by Holm. [2]Distributions were significantly different ('*': adjusted p-value<0.05, '**': adjusted p-value<0.01, "***': adjusted p-value<0.001).

Supplementary Table 16. Average methylation raters of spawning seasons by genomic regions.

Region	Methylation rate (%)				Order
	Off-season	Early	Normal	Late	
**Exon**	74.590	74.965	74.654	74.784	Off-season < Normal < Late < Early
**Intron**	85.142	85.412	85.524	85.193	Off-season < Late < Early < Normal
**P250**	21.923	22.299	22.087	21.755	Late < Off-season < Normal < Early
**P1K**	44.358	44.507	44.910	44.595	Off-season < Early < Late < Normal
**P5K**	76.011	76.230	76.363	75.994	Late < Off-season < Early < Normal
**Flank**	78.510	78.777	78.674	78.418	Late < Off-season < Normal < Early
**Misc**	83.891	84.232	84.320	84.099	Off-season < Late < Early < Normal
**IGR**	87.327	87.523	87.634	87.595	Off-season < Late < Early < Normal

Supplementary Table 17. List of genes with multiple DMCs in P250 and P1K between spawning seasons.

**Comp**[1]	**Gene ID**	**#DMCs (P250)**[2]			**#DMCs (P1K)**[3]			**Gene symbol**[4]	**Gene name**
		Total	Hypo	Hyper	Total	Hypo	Hyper		
**Off-season**	106579168	**3**	1	2	0	0	0	*klhl11*	kelch-like protein 11
	106609256	**3**	0	3	0	0	0	*prrx2*	paired mesoderm homeobox protein 2-like
	106583651	0	0	0	**4**	0	4	*helz2*	helicase with zinc finger domain 2-like
	106584059	0	0	0	**4**	0	4	*scp-2*	sterol carrier protein 2-like
	106563027	0	0	0	**3**	3	0	*slc31a1*	solute carrier family 31 member 1
	106581656	0	0	0	**3**	0	3	*lrrfip2*	leucine-rich repeat flightless-interacting protein 2-like
**Early**	106607889	**3**	0	3	0	0	0	*inf2*	inverted formin-2-like
	100196480	3	0	3	**4**	0	4	*ctl2b*	CTLA-2-beta
	106576160	0	0	0	**3**	0	3	*ttll12*	tubulin tyrosine ligase-like family, member 12
**Late**	106567512	0	0	0	**3**	2	1	*stk32a*	serine/threonine-protein kinase 32A
	106563027	0	0	0	**3**	3	0	*slc31a1*	solute carrier family 31 member 1
	106573279	0	0	0	**3**	0	3	-	Uncharacterized

[1]Pair-wise comparisons against the normal season. [2,3]Number of DMCs in P250 and P1K regions. Genes were selected when they had three or more DMCs either in P250 or P1K. Counts in bold face indicates genes with three or more DMCs. [4]When gene symbols were unavailable, we utilised the gene symbols from either the latest NCBI version or UniProt as substitutes.

Supplementary Table 18. List of DEGs with at least one DMC in P250 and P1K between spawning seasons.

Cmp[1]	Gene ID	LFC	Chr[2]	Start[3]	Reg	Mdf[4]	Symbol	Gene name[5]
**Off-season**	106562317	-0.72	NC_027310	24493341	P250	-21.97	*caprin-1*	caprin-1
	106599887	-0.69	NC_027302	32167196	P250	-18.86	*cyp8b1*	5-beta-cholestane-3-alpha,7-alpha-diol 12-alpha-hydroxylase
**Early**	106561604	-0.85	NC_027309	105304013	P1K	23.04	*aurkb*	aurora kinase B
	106570052	1.04	NC_027300	15370776	P1K	17.95	*lpin1*	phosphatidate phosphatase LPIN1
	106582038	-0.85	NC_027320	29797092	P1K	-16.58	*adrenodoxin*	adrenodoxin
	106600949 (*)	1.09	NC_027302	52643520	P1K	-18.40	-	ferritin, mid-dle subunit
			NC_027302	52643563	P1K	-26.45		
	106604665	0.99	NC_027304	30317427	P1K	-18.38	*slc43a1a*	solute carrier family 43 member 1a
	106588407	-1.00	NC_027326	9996221	P250	-27.36	*caprin-1*	caprin-1
**Late**	106561604	-0.73	NC_027309	105304013	P1K	25.66	*aurkb*	aurora kinase B
	106588407	-0.93	NC_027326	9996221	P250	-24.39	*kifc1*	carboxy-terminal ki-nesin 2

*: Gene ID 106600949 is discontinued in the current NCBI as of 2023. Hence, it is excluded from the main text.

[1]Pair-wise comparisons against the normal season. [2,3]Chromsome (Chr) and start position (Start) of DMCs. Suffix of chromosome ".1" was removed. For instance, the full name of NC_027310 is NC027310.01. [4]Mdf: methylation rate difference of DMC. [5]When gene symbols were unavailable, we utilised the gene symbols from either the latest NCBI version or UniProt as substitutes.

Figures

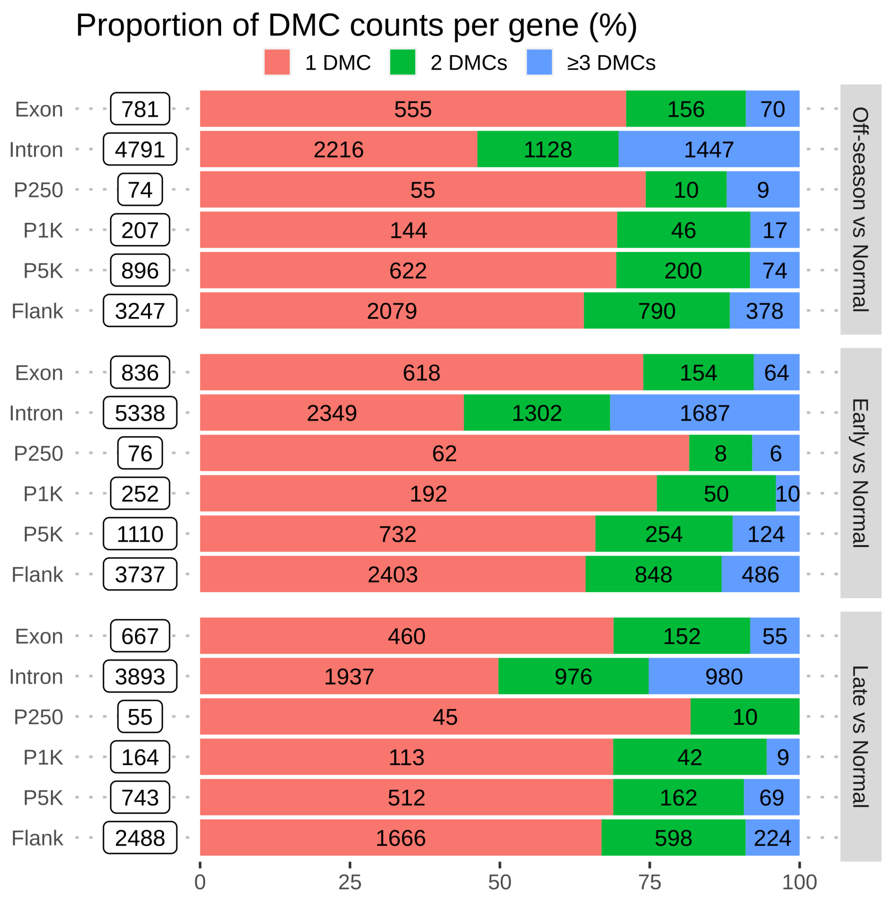

Supplementary Figure 1. DMC counts per gene by three pairwise comparisons.

Stacked barplots showing the number of DMCs per gene, 1 DMC (red), 2 DMCs (green), and greater than or equal to 3 DMCs (blue), in six different genomic regions. The x-axis represents proportions of DMC counts in percentage.
